# NSC-34 Motor Neuron-Like Cells Are Unsuitable as Experimental Model for Glutamate-Mediated Excitotoxicity

**DOI:** 10.3389/fncel.2016.00118

**Published:** 2016-05-09

**Authors:** Blandine Madji Hounoum, Patrick Vourc’h, Romain Felix, Philippe Corcia, Franck Patin, Maxime Guéguinou, Marie Potier-Cartereau, Christophe Vandier, Cédric Raoul, Christian R. Andres, Sylvie Mavel, Hélène Blasco

**Affiliations:** ^1^Institut National de la Santé et de la Recherche Médicale (INSERM U930) “Imagerie et Cerveau”, CHRU de Tours, Université François-RabelaisTours, France; ^2^Laboratoire de Biochimie et de Biologie Moléculaire, Hôpital Bretonneau, CHRU de ToursTours, France; ^3^Institut National de la Santé et de la Recherche Médicale (INSERM U1069) “Nutrition, Growth and Cancer”, Université François-Rabelais de ToursTours, France; ^4^Centre SLA, Service de Neurologie, CHRU de ToursTours, France; ^5^The Neuroscience Institute Montpellier, Institut National de la Santé et de la Recherche Médicale (INSERM UMR1051), Saint Eloi HospitalMontpellier, France

**Keywords:** ALS, Ca^2+^ influx, differentiation, NSC34, glutamate receptors, NMDA

## Abstract

Glutamate-induced excitotoxicity is a major contributor to motor neuron degeneration in the pathogenesis of amyotrophic lateral sclerosis (ALS). The spinal cord × Neuroblastoma hybrid cell line (NSC-34) is often used as a* bona fide* cellular model to investigate the physiopathological mechanisms of ALS. However, the physiological response of NSC-34 to glutamate remains insufficiently described. In this study, we evaluated the relevance of differentiated NSC-34 (NSC-34_D_) as an *in vitro* model for glutamate excitotoxicity studies. NSC-34_D_ showed morphological and physiological properties of motor neuron-like cells and expressed glutamate receptor subunits GluA1–4, GluN1 and GluN2A/D. Despite these diverse characteristics, no specific effect of glutamate was observed on cultured NSC-34_D_ survival and morphology, in contrast to what has been described in primary culture of motor neurons (MN). Moreover, a small non sustained increase in the concentration of intracellular calcium was observed in NSC-34_D_ after exposure to glutamate compared to primary MN. Our findings, together with the inability to obtain cultures containing only differentiated cells, suggest that the motor neuron-like NSC-34 cell line is not a suitable *in vitro* model to study glutamate-induced excitotoxicity. We suggest that the use of primary cultures of MN is more suitable than NSC-34 cell line to explore the pathogenesis of glutamate-mediated excitotoxicity at the cellular level in ALS and other motor neuron diseases.

## Introduction

Amyotrophic lateral sclerosis (ALS) is one of the most common neurodegenerative diseases in adults, caused by the selective death of motor neurons (MN). Studies of the physiology of ALS support the involvement of genetic factors and micro-environmental factors with mechanisms such as glutamate excitotoxicity and oxidative stress, but these mechanisms await elucidation. Glutamate excitotoxicity is a major contributor to dysfunction and death of MN in the pathogenesis of ALS (Heath and Shaw, 2002; Van Den Bosch et al., 2006; Spalloni et al., 2013; Blasco et al., 2014). Among the many drugs targeting this pathogenic mechanism tested in clinical trials (Blasco et al., 2014), one treatment (riluzole) is used in common practice to slow the progression of the disease by blocking glutamatergic neurotransmission in the central nervous system. However, the mechanisms of toxicity caused by glutamate and their links with other physiopathological pathways remain poorly understood.

Overstimulation of glutamate receptors facilitates the entry and consequently the excess of calcium (Ca^2+^) in cell compartments, leading to a cascade of destructive events by calcium-dependent enzymatic pathways and mitochondrial dysfunction with the generation of free radicals (Van Den Bosch et al., 2000; Blasco et al., 2014). Glutamate receptors are divided into two families: the ligand-gated cation channels (ionotropic; Lodge, 2009) and G protein-coupled receptors (metabotropic; Niswender and Conn, 2010). Ionotropic receptors are further divided into three categories according to their non-natural preferred agonists, i.e., AMPA (α-amino-3-hydroxy-5-methyl-4-isoxazole propionic acid), NMDA (*N*-methyl-D-aspartate) and kainate (AK; Lodge, 2009). AMPA and NMDA receptors are largely responsible for calcium flux across neuronal cell membranes (Hollmann et al., 1991; Van Den Bosch et al., 2000).

Primary cultures of MN (Krugman et al., 1999) and cultures of different cell lines are used to study the molecular mechanisms of neurotoxicity induced by glutamate in MN. The most common cell line used in ALS research is the spinal cord neuron × neuroblastoma hybrid cell line (NSC-34), which was originally described as having several morphological and physiological properties of MN (Eggett et al., 2000; Rembach et al., 2004; Benkler et al., 2013; Maier et al., 2013; Valbuena et al., 2015). We required validation of this model to study the metabolic effects of glutamate excitotoxicity in a neurological disease. NSC-34 is an hybrid cell line produced by the fusion of MN from the spinal cords of mouse embryos with mouse neuroblastoma cells N18TG2 (Cashman et al., 1992). These cells exhibit properties of MN when subjected to protocols of differentiation and maturation. Although NSC-34 cells share many features with MN such as long processes and S-laminin with leucine-arginine-glutamate (LRE) adhesion motif (a specific basal lamina glycoprotein concentrated at the neuromuscular synapse; Hunter et al., 1991), the formation of contacts with myotubes in culture (Cashman et al., 1992) and increased survival in the presence of neurotrophic factors (NTFs; Turner et al., 2004), their functional properties when stimulated by glutamate have rarely been studied (Durham et al., 1993; Eggett et al., 2000).

The selective motor neuronal cell death in ALS is highly dependent on intracellular Ca^2+^ and is insensitive to inhibitors of voltage-operated Ca^2+^ and Na^+^ channels (MacDermott et al., 1986; Van Den Bosch et al., 2000, 2006; Heath and Shaw, 2002). Thus, the permeability of glutamate receptors to Ca^2+^ seems crucial to validate the NSC-34 model as appropriate for the exploration of glutamate excitotoxicity. In order to evaluate whether NSC34 represents a relevant model for studies on glutamate-induced neurotoxicity, we differentiated the NSC-34 cells by serum depletion in the presence or absence of all-trans retinoic acid (RA; Clagett-Dame et al., 2006; Maden, 2007), and analyzed the expression and functionality of glutamate receptors. We compared for the first time the usefulness of NSC-34 cells (using various differentiation protocols) and primary motor neuron cultures (used as the reference) as models for glutamate induced-excitotoxicity in the same study.

## Materials and Methods

### Materials

Dulbecco’s modified Eagle’s medium (DMEM, Gibco), DMEM-Ham’s F12 and Alpha-modified Eagle’s medium (α-MEM), modified Eagle’s medium non-essential amino acid (MEM-NEAA), penicillin/streptomycin (P/S), Trizol, Superscript II reverse transcriptase kit and Fura-2 acetoxymethyl ester were all obtained from Invitrogen (Life Technologies, Saint Aubin, France), L-glutamic acid and *all-trans* RA from Sigma Aldrich (Saint-Quentin Fallavier, France) and fetal calf serum (FCS) from Eurobio (Courtaboeuf, France).

### Culture of NSC-34

The NSC-34 was obtained from Cedarlane Laboratories (*via* Tebu-Bio, Le Perray en Yvelines, France). Cells were cultured as described previously (Madji Hounoum et al., 2015). Cultures were used 5–15 passages. Each type of experiment was performed on the same passage. No sub-culture passage was performed for differentiated NSC-34.

For differentiation, NSC-34 cells were grown to confluence and the proliferation medium (DMEM plus 10% FCS) was exchanged for fresh differentiation medium every 3 days. Cells were allowed to differentiate for up to 4 weeks. Three differentiation media were investigated: (1) 1:1 DMEM/Ham’s F12 plus 1% FCS, 1% P/S and 1% MEM-NEAA, the most commonly used medium for NSC-34 differentiation (Kruman et al., 1999; Eggett et al., 2000; Rembach et al., 2004; Benkler et al., 2013); (2) α-MEM [the medium used for another neuron-like cell line (P19; MacPherson et al., 1997)] plus 1% FCS, 1% P/S and 1% MEM-NEAA; and (3) DMEM (the classic medium for NSC-34 culture) plus 1% FCS and 1% P/S.

Two conditions were investigated for all differentiation media, i.e., with or without RA [1 μM, as used previously (Johann et al., 2011; Maier et al., 2013)]. NSC-34 cells, maintained on proliferation medium, served as the undifferentiated control group. The average length of neurites in the differentiation media was quantified using Sholl’s method for quantification of dendritic branching in hippocampal neurons (Sholl, 1956). Briefly, concentric circles at 25 μm intervals between adjacent circles were drawn on Powerpoint, at the same magnification of cell pictures. The center of the circle centered on the soma of the cell, the lengths of the processes were measured from the soma by multiplying the number of intersections (neurite-circle) every 25 μm. Cells with neurites longer than 50 μm were considered as differentiated. Neurite length was analyzed by imaging a minimum of 10 cells per experiment, four experiments for each condition.

### Primary Motor Neuron Cultures

Studies were conducted using primary cultures of motor neuron from the spinal cords of C57BL/6 mice at embryonic day 12.5 (Centre d’Elevage Roger Janvier, France). Cultures were grown as described previously (Camu et al., 2014; Dangoumau et al., 2015). MN were plated on poly-ornithine/laminin-treated wells in the presence of NTFs (0.1 ng/mL GDNF, 1 ng/mL BDNF, and 10 ng/mL CNTF in supplemented neurobasal medium (Invitrogen, Carlsbad, CA, USA)). Supplemented neurobasal medium contained 2% horse serum, L-glutamate (25 mM), β-mercaptoethanol (25 mM), L-glutamine (0.5 mM), and 2% B-27 supplement (Invitrogen, Life Technologies, Saint Aubin, France). The use of appropriate culture medium combined with multiple purification steps using density gradient (BSA cushion and Optiprep density centrifugation) and magnetic cell sorting with an indirect microbeads technique promoted the enrichment of MN as well as the elimination of astrocytes and microglia cells from the culture (Arce et al., 1999).

### Immunocytochemistry

To assess morphological characterization of primary motor neuron culture derived from embryonic spinal cord, expression of βIII-tubulin and p75 neurotrophic receptor were analyzed (Rembach et al., 2004). Cells were fixed in 4% paraformaldehyde and then incubated in a blocking and permeabilizing solution (10% donkey serum and 2% Triton X-100) for 1 h. The cells were stained with rabbit anti-βIII-tubulin (1:200, Covance, Princeton, NJ, USA) and mouse anti-nerve growth factor receptor (p75^NTR^ 1:60, Chemicon MAB357) for 1 h. After being washed with phosphate buffered saline (PBS), the cells were incubated with secondary antibodies for 1 h. The secondary antibodies used were donkey anti-rabbit Alexa-488 (1:300, Life Technologies, Saint Aubin, France) and donkey anti-mouse Alexa-594 (1:300, Life Technologies, Saint Aubin, France). Nuclear DNA was observed by 4,6-diamino-2-phenylindole (DAPI) contained in ProLong^®^ (Life Technologies, Saint Aubin, France). Cells were photographed using a confocal microscope (Olympus FV500).

### Cell Viability Assay

To assess the effect of glutamate-induced excitotoxicity in NSC-34_D_, cells were exposed to glutamate at different concentrations (100 μM, 500 μM, 1 mM and 10 mM) for 48 h (Eggett et al., 2000; Rembach et al., 2004; Maier et al., 2013) before determination of cell viability by trypan blue assay. A control condition with no excitotoxic agent (PBS) was included for comparison. Four replicates per condition were obtained. The cells were harvested with trypsin and re-suspended in culture medium before addition of trypan blue (0.4% w/v; Molecular Probes-Invitrogen, Carlsbad, CA, USA). Living and dead cells were counted accurately with a Countess Automated Cell Counter (Invitrogen, Carlsbad, CA, USA). Motor neuron survival was assessed by direct counting after exposing cells to 100 μM glutamate for 48 h.

### RNA Extraction, Reverse Transcription PCR (RT-PCR) and Real-Time Polymerase Chain Reaction (RT-qPCR)

Following a wash in PBS, RNA samples were extracted from NSC-34 cells and primary MN using Trizol and treated with DNase I (Proteigene^®^). Reverse transcription was performed on 2.5 μg of treated RNA using the superscript II reverse transcriptase kit according to the manufacturer’s instructions. cDNA samples were analyzed using primers for subunits of AMPA receptors (GluA1–4), NMDA receptors (GluN1, GluN2A-D, GluN3A), and standard markers for MN such as choline acetyltransferase (ChAT), p75 (Cashman et al., 1992; Matusica et al., 2008; Maier et al., 2013; see Table A1 in Supplementary Material). Actin was used as internal standard. cDNA samples were amplified using GoTaq^®^ Flexi DNA polymerase (Promega) in 25 μL reaction mixture containing 125 ng cDNA following the manufacturer’s protocol. Amplification consisted of 34 cycles at 95°C for 1 min, 59°C for 1 min and 72°C for 1.5 min. Aliquots of 10 μL of these products underwent electrophoresis on 1.5% agarose gel before capture with a ChemiDoc XRS camera and quantification by Quantity One Software (BioRad). Mouse brain extract was used as positive control for amplifications.

The gene expression of glutamate receptor subunits, ChAT and p75^NTR^, was measured by semi quantitative real-time PCR (RT-qPCR) using the Brilliant III Ultra-Fast SYBR^®^ QPCR MM kit (Agilent^TM^) and the same primers as for conventional RT-PCR (see Table A1 in Supplementary Material). The reaction was performed in a LightCycler 480 (Roche Diagnostic, Meylan, France) at Tm 60–62°C. The efficiency of amplification was calculated on cDNA at concentrations ranging from 3.75 to 60 ng/mL. To ensure the absence of genomic DNA contamination, a control sample of non-reverse-transcribed RNA was run for each set of RNA extractions. The expression’s stability of reference genes was determined following MIQE guidelines (Bustin et al., 2009). We showed that β-actin and GAPDH genes were stably expressed (data not shown). Relative quantification was obtained by calculating the ratio between the values obtained for each gene of interest and the reference genes (β-actin, GAPDH). Melting curves were routinely performed to determine the specificity of the qPCR reaction. The 2^−ΔΔCt^ method was used for analysis.

### Measurement of Calcium Influx

Cells were seeded on coverslips at a density of 3 × 10^4^ cells/mL and allowed to grow for 2 days. They were loaded with a fluorescent probe (1 μM Fura-2 AM) in culture medium for 45 min at 37°C. Cells were then washed with medium and allowed to de-esterify for at least 15 min at room temperature. The dish was then placed on the stage of a Nikon Eclipse TE2000-S inverted fluorescence microscope (Nikon, France) equipped with a 75 W Xenon Arc Lamp (Ushio, Japan), an Optoscan Monochromator (Cairn Optoscan, Kent, UK), and an ORCA-03G (CCD) camera (Hamamatsu, Japan). The coverslips were placed in a perfusion chamber containing physiological solution with 2 mM calcium (see Table A2 in Supplementary Material).

Excitation light was chopped by the monochromator at the two excitation wavelength maxima of fura-2 (340/380 nm). The excitation protocol used was a 500 ms excitation at each wavelength every 4 s. Fluorescence emission at 510 was detected by the CCD digital camera. The software (Imaging workbench 6) excited cells and allowed acquisition of images. The acute effects of glutamate at 100 μM and 1 mM (for primary MN and NSC-34_D_, respectively) on calcium entry were measured in physiological solution for 200 s. For primary motor neuron cells, we first determined the optimal time window allowing the experiment by measuring calcium entry every 2 days on independent replicates (cells that had not been exposed to fura-2 experiment before).

### Statistical Analysis

Data was expressed as the mean ± SEM values. Statistical significance was determined using the Mann-Withney test or one-way Anova. For multiple comparisons, Tukey’s tests were used as *post hoc* tests or Kruskal-wallis test for appropriate analyses. For all analyses, *p* values < 0.05 were considered significant. The type of statistical test is specified in each figure legend.

## Results

### Efficient Differentiation of NSC-34 Cells into Motor Neuron-Like Cells

**Figure 1:**
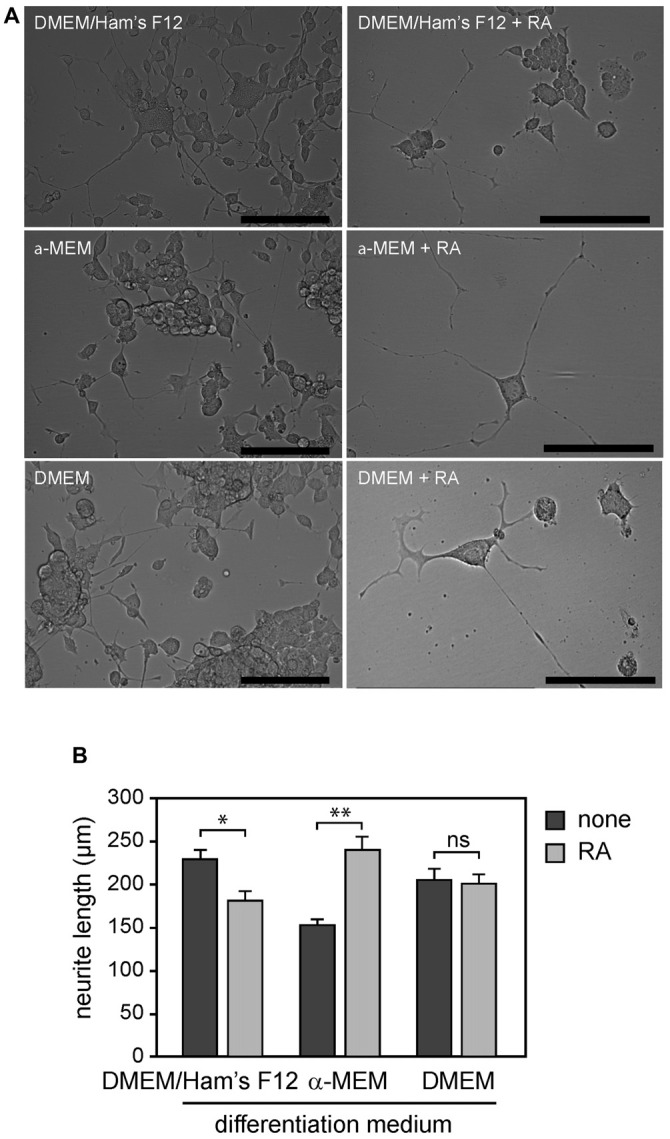
**(A)** NSC-34 cells grown for 4 weeks in differentiation media with or without addition of *all-trans* retinoic acid (RA): DMEM/Ham’s F12, α-MEM and DMEM. **(B)** Morphological differentiation of NSC-34 cells determined by measurement of neurite length. Statistically significant difference from differentiated cells without addition of RA was defined using Mann-Whitney test, according to the different media: DMEM/Ham’s F12, α-MEM and DMEM. Values are means ± SEM (10–11 cells per experiment and four experiments for each condition), *p < 0.001, **p < 0.0001, ns = not significant, Mann-Whitney test + RA vs. none.

When NSC-34 cells were cultured in the differentiation media (NSC-34_D_), we distinguished two morphologically distinct populations: cells with short neurites, and cells with phenotypic characterization of MN with long processes (Figure 1A). However, this differentiation was preceded by a massive loss of cells (>50%) as described by Eggett et al. (2000), with surviving cells sprouting extensive neuron-like projections.

We next investigated whether the potent morphogen RA could enhance differentiation of NSC-34 into motor neuron-like cells. We found that addition of RA (1 μM) in differentiation medium resulted in a significant decrease (60–70%) in proliferation at 4 days. Surviving cells showed a very low rate of proliferation compared to conditions without RA, as previously shown (Johann et al., 2011). However, we observed that a significant proportion of surviving cells had a motor neuron-like phenotype (~80%) in the presence of RA as determined by morphological criteria (Figure 1A).

We then assessed whether differentiation conditions could potentiate the effects of RA on process outgrowth of NSC-34 cells. We determined the average neurite length of NSC-34 cells when they are maintained for 4 weeks in three differentiation media (α-MEM, DMEM/Ham’s F12 and DMEM) in the presence or absence of RA. We found that the average neurite length has increased by 55% when they are cultured in α-MEM media with RA compared to α-MEM media without RA (240.00 ± 15.89 and 154.16 ± 7.01, respectively, *p* < 0.0001). While it has decreased by 20% when cells were cultured in DMEM/Ham’s F12 media with RA compared to media without RA (181.82 ± 11.29 and 229.69 ± 12.09, respectively, *p* < 0.001; Figure 1B). We did not find any significant change when NSC-34 cells were cultured in DMEM with or without RA (200.45 ± 11.95 and 206.25 ± 13.13, respectively, *p* = 0.46). Therefore, the effects of RA on NSC-34_D_ cell morphology were highly dependent on the differentiation medium (Figure 1B).

### Differentiated NSC-34 Cells Expressed Glutamate Receptor Subunits

**Figure 2:**
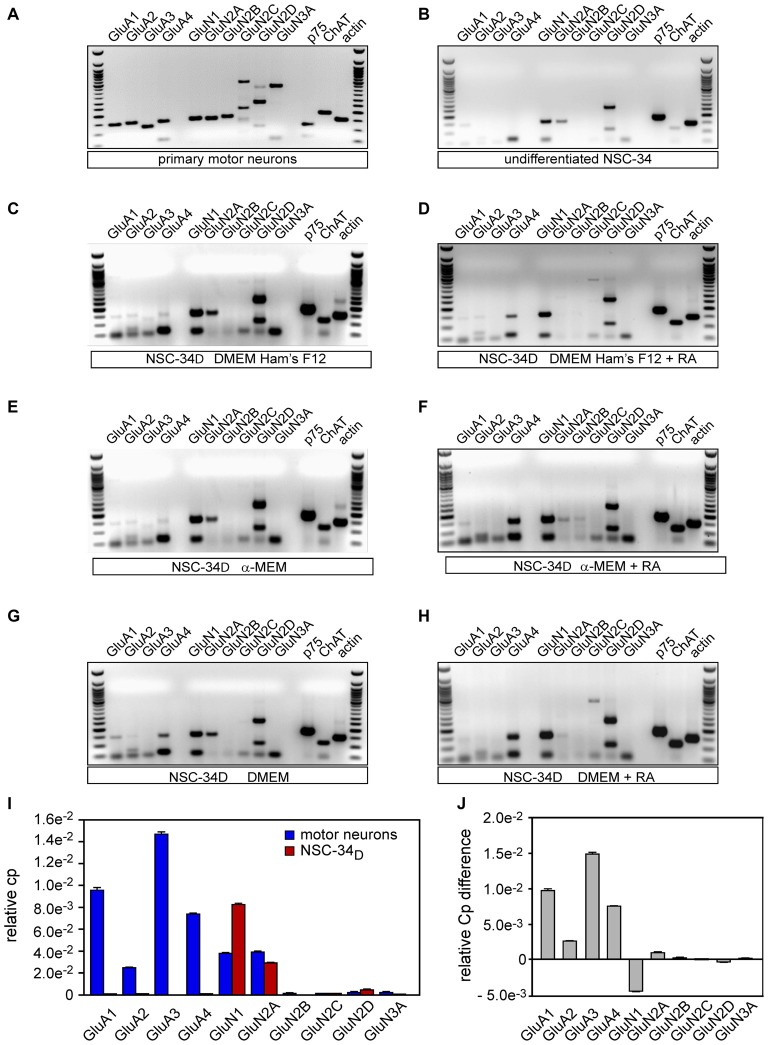
**Characterization of the expression of glutamatergic receptors in motor neurons (MNs) and NSC-34_D_ cells.** Conventional RT-PCR on RNA isolated from NSC-34 cells maintained in the proliferation medium (undifferentiated) **(A)**, from MNs **(B)**, and from NSC-34 cells grown in three differentiation media with or without *all trans*-retinoic-acid (AR) for 4 weeks: **(C,D)** DMEM/Ham’s F12, **(E,F)** MEM and **(G,H)** DMEM. **(I)** Semi-quantitative RT-qPCR analysis of gene expression of glutamate receptor subunits in MN and NSC-34 differentiated in DMEM/Ham’s F12 media without RA (mean ± SEM, *n* = 3). **(J)** Semi quantification of the differences in RT-qPCR between MN and NSC-34_D_ in DMEM/Ham’s F12 media without RA (mean ± SEM, *n* = 3).

We first set up conditions for optimal and specific amplification of glutamate receptor subunits GluA1–4, GluN1, GluN2A-D and GluN3A using total RNA obtained from mouse brain extract (see Figure A1 in Supplementary Material). We next studied the expression profiles of all glutamate receptors promoted by NSC-34 differentiation (up for 4 weeks) and in primary MN. We found that the differentiation of NSC-34 in the conditions that we had previously described induced a significant increase in mRNA expression of glutamate receptor subunits GluN1, GluN2A and GluN2D compared to undifferentiated cells (Figures 2B–I). The example of GluN2A protein confirms this statement: the result obtained by western blot analysis showed that the GluN2A protein level in NSC-34_D_ was higher than in undifferentiated NSC-34 (see Figure A2 in Supplementary Material). Levels of GluA4 subunits mRNA have increased in DMEM without RA compared to the other conditions without RA (Figures 2C,E,G). At more discrete levels, we also observed increases of GluA2 and GluA3 mRNA in DMEM/Ham’s F12 and α-MEM but not in DMEM. We noted the expression of all glutamate subunits in primary MN, as observed in mouse brain extracts (Figure 2A and Figure A1, see Supplementary Material).

We found that the presence of RA promoted the expression of GluA4 mRNA in every differentiation medium (Figures 2C–H). However, mRNA expression of the other subunits of glutamate receptors was not modified by the presence of RA in the culture medium. The differentiation condition that promoted most of the glutamate receptors and that generated less heterogeneity bias was thus DMEM-Ham’s F12 without RA. Indeed, this differentiation condition led to expression of all AMPR subunits (GluA1–4) and half of the NMDAR subunits (GluN1, GluN2A, and GluN2D), as summarized in Table A3 (see Supplementary Material). However, expression of AMPR subunits was lower compared to primary MN (Figures 2A,C,I,J). DMEM-Ham’s F12 without RA medium is used for further explorations. Our results also showed that NSC-34_D_ cells expressed many motor neuron properties such as ChAT and p75^NTR^ (Figures 2C–H). RA-differentiated NSC-34 did not express more receptor subunits than NSC-34_D_ without RA (Figures 2C–H and Table A3 in Supplementary Material). We next semi quantified expression of glutamate receptor subunits in NSC-34_D_ differentiated in DMEM-Ham’s F12 without RA condition compared to MN.

### Expression of Receptor Subunits

RT-qPCR analysis revealed high levels of expression of GluN1, GluN2A and GluN2D mRNA (CP values were 8.35 × 10^−3^ ± 4.96 × 10^−5^, 3.02 × 10^−3^ ± 6.30 × 10^−8^ and 4.95 × 10^−4^ ± 1.83 × 10^−6^, respectively) in NSC-34 differentiated in DMEM/Ham’s F12 without RA, while expression of GluA1, GluA2, GluA4, GluN2C and GluN3A mRNA (9.85 × 10^−7^ ± 4.02 × 10^−9^, 4.45 × 10^−7^ ± 5.78 × 10^−9^, 7.45 × 10^−6^ ± 5.77 × 10^−9^, 3.22 × 10^−6^ ± 1.80 × 10^−7^ and 9.74 × 10 ^−6^ ± 2.01 × 10^−8^, respectively) was low in NSC-34_D_ (Figures 2I,J), i.e., as for the results of conventional RT-PCR (Figure 2C). GluA3, and GluN2B mRNA levels were almost zero in NSC-34_D_ (Figures 2I,J). It is of note that only 20% of the cells did not express motor neuron-like morphology. The results showed that primary MN expressed all glutamate receptor subunits at different levels, unlike to NSC-34_D_. Consistently with RT-PCR data, the transcript profile of NSC-34_D_ glutamate receptor subunits is different from that of MN.

### Differentiated NSC-34 Cells did not Show Susceptibility to Glutamate-Induced Death

**Figure 3:**
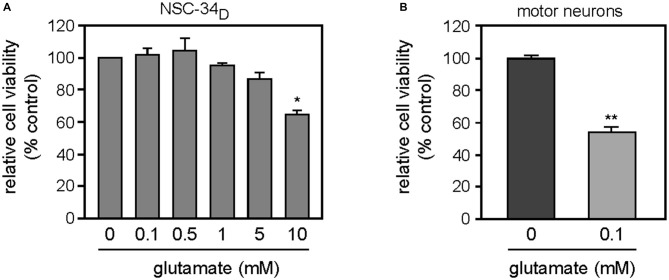
**(A)** Effects of glutamate on NSC-34_D_ cell cultured in DMEM/Ham’s F12 without RA. Cells were exposed to increasing concentrations of glutamate (ranging from 0.1 to 10 mM) for 48 h before counting. **(B)** Effects of glutamate on survival of primary MN, cells were exposed at 8 DIV to 0.1 mM glutamate for 48 h before counting. Data were analyzed by Mann-Whitney test or Kruskal-Wallis (multiple comparisons) to detect statistical significance. Statistically significant difference from untreated cells *p < 0.05, **p < 0.01. Data are mean ± SEM (*n* = 3).

We investigated whether glutamate could trigger death of differentiated NSC-34 cells. NSC-34_D_ cells were exposed to increasing concentrations of glutamate ranging from 0.1 to 10 mM. We found 102.04 ± 3.63%, 104.08 ± 8.03%, 94.90 ± 1.44%, 86.73 ± 4.17% and 64.28 ± 2.89% of relative cell viability when cells exposed to 0.1 mM, 0.5 mM, 1 mM, 5 mM and 10 mM glutamate, respectively (Figure 3A). We also evaluated excitotoxicity death of MN at 8 DIV (Days *In Vitro*) exposed to 0.1 mM glutamate and we found 53.99 ± 3.04% of relative cell viability (Figure 3B). We observed that 48 h exposure to 0–5 mM glutamate did not elicit any toxic response to NSC-34_D_, and a non-specific effect of about 30% on cells survival was found when glutamate was added at 100-fold higher concentrations (10 mM; Figure 3A) than that necessary to induce 50% death of primary MN (0.1 mM; Figure 3B). The results show that NSC-34_D_ cells do not show the same susceptibility to glutamate-induced excitotoxicity compared to MN.

### Glutamate did not Elicit Sustained Calcium Entry into NSC-34_D_

Glutamate-induced excitotoxicity resulted in elevated intracellular calcium levels *via* ionotropic glutamate receptors and activation of cell death signaling pathways. To address the effects of glutamate on Ca^2+^ transients in NSC-34_D_, we used ratiometric Ca^2+^ measurement with the imaging dye Fura-2AM. To establish sensitive and accurate measurement of intracellular Ca^2+^, we used motor neuron-enriched cultures. When MN were purified from E12.5 embryos as described above, a culture containing about 70% MN was identified using the p75^NTR^ generic marker (Figure A3-A see Supplementary Material). The phase-bright soma with long motor neuron processes made them clearly distinguishable from other cells (Figure A3 see Supplementary Material). Their maturity was tested every 2 days by measuring [Ca^2+^] influx to determine the optimal time window allowing the experiment. We found that younger MN did not efficiently load Fura-2AM before 5 DIV (data not shown). We observed that MN showed robust fluorescence signal from 5 DIV and glutamate induced a robust Ca^2+^ influx in MN at 12 DIV (Figure A3-B, see Supplementary Material). The maximum fura-2 fluorescence intensity relative to baseline was significantly higher (*p* < 0.005) in MN at 12 DIV (0.0293 ± 0.0026) and 13 DIV (0.0320 ± 0.0004) compared to those at 5 DIV (0.0019 ± 0.0002). There was no significant difference between MN at 12 DIV and 13 DIV. Subsequent experiments were therefore performed using 13-day-old cultures (Figure A3-B, see Supplementary Material). At 13 DIV, the MN are fully mature with electrical activity (Jackson et al., 1982; Nicola et al., 1992; Chang and Martin, 2011).

**Figure 4:**
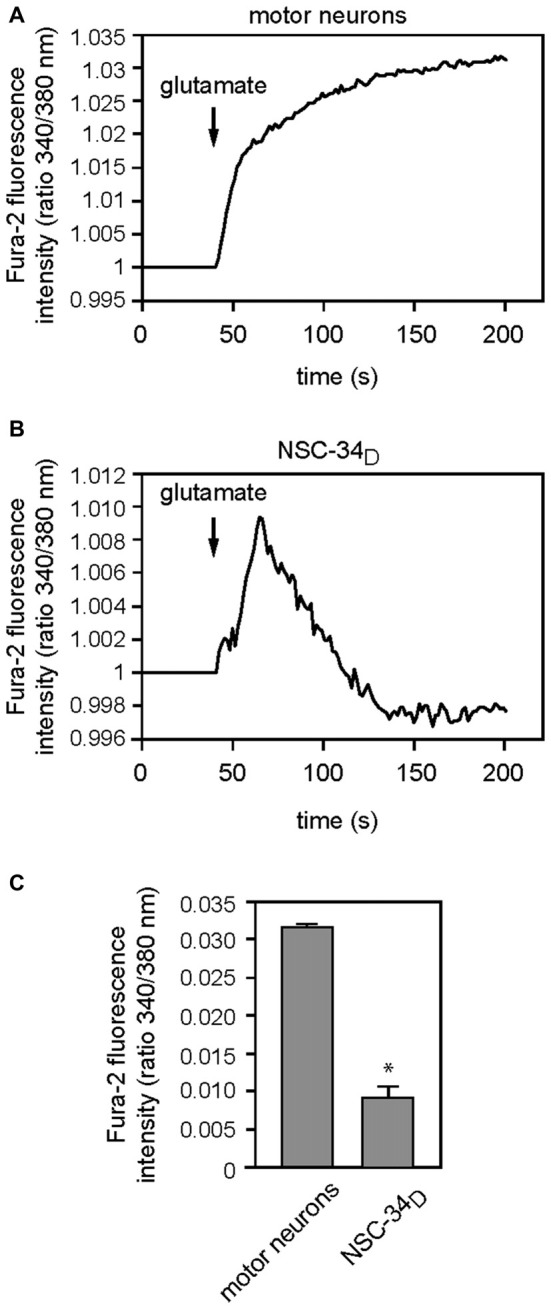
**Acute effects of 100 μM and 1 mM glutamate (for primary MN and NSC-34_D_, respectively) for 200 s on calcium entry.** Representative fluorescence measurement of Ca^2+^ entry in MN **(A)** and NSC-34_D_ **(B)** after acute application of glutamate. Glutamate was added at the time indicated by the arrow. **(A)** Mean traces of cytosolic Ca^2+^ flux of MN and **(B)** NSC-34_D_ in response to glutamate. **(C)** Histograms showing maximum fluorescence intensity following glutamate application in MN and NSC-34_D_. Mean data ± SEM from three coverslips per cell type with at least five cells per coverslip analyzed. *p < 0.001 (Mann-Whitney test MN vs. NSC-34_D_).

We then investigated the functionality of glutamate receptors expressed in NSC-34_D_ growing in DMEM/Ham’s F12 medium without RA for 4 weeks through Ca^2+^ permeability. The baseline recordings (first 0.5 min in all trace graphs) demonstrate stable cytosolic calcium levels in both MN and NSC-34_D_ cells before treatment with glutamate. The addition of glutamate immediately increased the fluorescence intensity of the calcium-sensitive dye Fura-2, indicating increased cytosolic Ca^2+^ flux in MN (Figure 4A). Whereas MN presented a sustained calcium entry following acute stimulation by glutamate (0.1 mM), glutamate stimulation in NSC34_D_ resulted in a small transient calcium entry (Figure 4B). However, in the presence of glutamate, Ca^2+^ entry into MN was significantly higher than in NSC-34_D_ cells, and the signal was as consistent as that obtained by fura-2 fluorescence intensity relative to baseline (0.032 ± 0.0004 vs. 0.009 ± 0.0012; *p < 0.001; Figure 4C). Taken together, these results showed that an increased extracellular glutamate concentration (1 mM) did not elicit the same calcium response in NSC-34_D_ cells (Figure 4C) as in MN.

## Discussion

Although glutamate-mediated excitotoxicity is known to be involved in ALS pathogenesis (Beal, 1992; Mattson et al., 1992), the precise mechanisms leading to electrical, metabolic or signaling dysfunction in the motor neuron diseases remain to be determined. Excitotoxicity mechanisms have been described following *in vitro* studies using either primary motor neuron cultures (Kruman et al., 1999; Van Den Bosch et al., 2000; Dolga et al., 2011; Joshi et al., 2011) or cell lines (Rembach et al., 2004; Benkler et al., 2013). The advantage of primary cultures is the development of similar *in situ* counterpart morphology and physiology over time. The neurotoxicity of a variety of toxins *in situ* has also been reported (Durham et al., 1993). However, due to the low yields of motor neuron cultures, the NSC-34 cell line has been widely used and tacitly considered to be the most stable motor neuron cell line to model ALS pathophysiology (Veyrat-Durebex et al., 2014). Indeed, many studies have shown that the differentiation process can induce NSC-34 cells to express certain motor neuron properties and have shown some toxic effects following exposure to glutamate (Eggett et al., 2000; Benkler et al., 2013; Maier et al., 2013). The differentiation and the maturation of NSC-34 cells are generally characterized by extension of neurites and expression of certain motor neuron-specific proteins. In this study, we investigated the reliability of these cells as an *in vitro* model to study glutamate-mediated neurotoxicity, using an approach from receptor expression to the Ca^2+^ influx.

### Differentiation of NSC-34 Cells into Motor Neuron-Like Cells

Differentiation of cell lines frequently requires modification of the culture medium through serum depletion and/or and use of chemical reagents or metabolites such as RA to obtain more neuron-like properties, including neurite outgrowth and morphological changes (MacPherson et al., 1997; Eggett et al., 2000; Maier et al., 2013). As reported in the literature, we found that the differentiation process induced NSC-34 cells to express phenotypic MN with long processes (Figure 1A and Figure A3-A, see Supplementary Material). We found that the RA effects on NSC-34_D_ cell morphology were highly dependent on the differentiation medium (Figure 1B). Importantly, we did not observe any effect on undifferentiated NSC-34 cells as previously demonstrated (Cashman et al., 1992; Maier et al., 2013). We also evaluated the RA effects on the expression of genes encoding certain specific motor neuron proteins and glutamate receptor subunits, and we found that RA-differentiated NSC-34 did not express more receptor subunits than NSC-34_D_ without RA (Figures 2C–H and Table A3, see Supplementary Material), thus suggesting that using RA is not required in NSC-34 differentiation processes in the context of excitotoxicity studies. This conclusion is supported by the results obtained by Cheung et al. (2009) who showed that RA differentiation conferred higher tolerance of SH-SY5Y cells to neurotoxin (6-hydroxydopamine) compared to undifferentiated SH-SY5Y cells, and they suggested that RA-differentiated SH-SY5Y was not appropriate for neurotoxicity or neuroprotection studies.

### The Receptor Subunits Expressed by NSC-34_D_

NMDARs and AMPARs have critical roles in excitatory synaptic transmission, plasticity and excitotoxicity in the CNS. We found that NSC-34_D_ expressed more receptor subunits in DMEM/Ham’s F12 medium without RA than in other media (Figures 2C–H and Table A3, see Supplementary Material). NSC-34_D_ expressed all AMPAR (GluA1–4) and some NMDAR (GluN1, GluN2A/B) subunits, as reported in the literature (Eggett et al., 2000; Rembach et al., 2004). We therefore chose DMEM/Ham’s F12 medium without RA for further experiments. Then we compared expression of glutamate receptor subunits on primary MN and NSC-34_D_ using RT-qPCR (Figures 2I,J). The results showed that primary MN expressed all glutamate receptor subunits at different levels, in contrast to NSC-34_D_ which expressed some of them at times and in very small quantities. This clearly showed that the transcript profile of NSC-34_D_ glutamate receptor subunits was different from that of MN and very consistent with RT-PCR and RT-qPCR data, indicating that NSC-34_D_ could not be used as a model of primary MN for glutamate-mediated excitotoxicity.

### No Glutamate-Induced Toxicity in NSC-34_D_

Glutamate concentrations in the extracellular fluid are normally around 0.8–2.9 μM (Lerma et al., 1986). A rise in the extracellular glutamate concentration to 2–5 μM is considered sufficient to cause degeneration of neurons through excessive stimulation of glutamate receptors (Rosenberg et al., 1992). When investigating glutamate-induced excitotoxicity at the cellular level, the glutamate concentrations used to obtain effects varied according to the *in* *vitro* models investigated and exposure times. For primary cultures, the range of glutamate concentrations used to act on ionotropic glutamate receptors (Van Den Bosch et al., 2000) was between 10 μM–1 mM, and cultures were exposed for less than 24 h (Mattson et al., 1995; Kruman et al., 1999; Sen et al., 2008). As in our study (Figure 3B), it had previously been demonstrated that exposure of primary embryonic MN to glutamate (0.1 mM) for 24 or 48 h led to approximately 50% cell death (Metzger et al., 1998; Urushitani et al., 1998), whereas for cell lines, this glutamate concentration range was increased to 10 mM with longer exposure times (24–72 h; Eggett et al., 2000; Rembach et al., 2004; Benkler et al., 2013; Maier et al., 2013). In our study, we investigated the toxicity of glutamate on NSC-34_D_ and motor neuron survival. The results showed approximately 50% cell death at 100 μM glutamate for primary MN (Figure 3B), as described in the literature (Metzger et al., 1998; Urushitani et al., 1998). For NSC-34_D_ cells, we found a significant loss of viable cells at a concentration of only 10 mM glutamate (Figure 3A). This result is consistent with previous excitotoxicity studies on NSC-34_D_ where treatment with glutamate (up to 1 mM) failed to induce vacuolation of NSC-34_D_, in contrast to the effects on primary MN (Durham et al., 1993).

Among the different neuroprotective mechanisms in neurons, neurotrophin factors may play a key role. Literature reported that NTFs and glutamate interact to regulate developmental and adult neuroplasticity (Mattson et al., 1995; Mattson, 2008). Consequently, neurotrophin factors attenuated elevation of intracellular calcium concentrations induced by glutamate (Mattson et al., 1995), thus promoting the survival of neurons (McKay et al., 1996; Giménez y Ribotta et al., 1997; Vincent et al., 2004; Mattson, 2008) via the MAPK and PI-3K/Akt pathways (Vincent et al., 2004).

To validate the glutamate-sensitive motor neuron NSC-34 cell line, many studies have used modified growth conditions through serum depletion to induce differentiated NSC-34 cells and to investigate the cytotoxicity of glutamate or other excitotoxins (AMPA, 5-fluorowillardiine, H_2_O_2_, TNF-α, *etc*.) in the presence or absence of receptor antagonists by using viability tests (Rembach et al., 2004; Hemendinger et al., 2012; Benkler et al., 2013; Maier et al., 2013). In some cases, they evaluated the expression of certain genes which encode cholinergic phenotype-related proteins (ChAT, acetylcholine esterase and vesicular acetylcholine transferase; Maier et al., 2013). It should be emphasized that most studies that used cell line models to investigate glutamate neurotoxicity focused predominantly on its toxic action and not on the functional properties of glutamatergic receptors such as Ca^2+^ permeability (Rembach et al., 2004; Hemendinger et al., 2012; Benkler et al., 2013; Maier et al., 2013).

### No Sustained Calcium Entry in NSC-34_D_

The stoichiometry of the receptor complexes in mammalian cells seems to be largely controlled by the level of the individual subunits expression that determine their functional properties including Ca^2+^ permeability *via* glutamate receptors (Hollmann et al., 1991; Monyer et al., 1992; Das et al., 1998; Dingledine et al., 1999; Sobolevsky et al., 2009). According to the literature evidence, the expression of GluA1–4, GluN1 and GluN2A or GluN2B subunits induces functional receptors. Surprisingly, in our study, despite the expression of GluA, GluN1 and GluN2A/D subunits in NSC-34_D_ (Figure 2I), we did not observe a significant sustained calcium entry as demonstrated in MN (Figure 4C). In this study, we globally evaluated the intracellular calcium concentrations (including the interplay between extracellular and intracellular origin) provoked by glutamate, through glutamate receptors by using the Fura-2-AM. However, we cannot exclude the possibility that part of the increase of cytosolic calcium could come from internal stores. Using SH-SY5Y cells line and measurement of calcium release from different compartments, Jaiswal et al. (2009) provided evidences of the existence of two separate intracellular Ca^2+^ stores (endoplasmic reticulum (ER) and mitochondrial intracellular pools) which may participate in the generation of intracellular Ca^2+^ signals. Calcium permeability *via* glutamate receptors has also been shown to initiate a self-perpetuating process of intracellular Ca^2+^ dysregulation with consecutive ER Ca^2+^ release and mitochondrial Ca^2+^ overload (Jahn et al., 2006; Grosskreutz et al., 2007, 2010). The ER and mitochondria form a highly dynamic interconnected network that is involved in the generation of Ca^2+^ signals (Tadic et al., 2014). A recent article reported a Ca^2+^ influx in NSC-34_D_ cells compared to a “negative control” undifferentiated NSC-34 (Liu et al., 2015), but authors did not compare calcium influx of these NSC-34_D_ to that of MN (as “positive control”) which are the parent motor neuron for NSC-34, as performed here in our study. We postulate that this shift of Ca^2+^ entry in NSC-34_D_ could be due to the fact that some glutamate receptor subunits were not expressed or very weakly expressed, in contrast to MN which showed a sustained calcium entry following glutamate application.

### Limitations in the Use of the NSC-34 Cell Line

As mentioned above, Eggett et al. (2000) evaluated the use of NSC-34 cells as a glutamate-sensitive motor neuron model. Using immunocytochemistry, they demonstrated the presence of glutamate receptor proteins GluN1, GluN2A/B, GluA1–4, GluK2/3 and GluK5. Exposure to glutamate (1 mM) for 24 h induced significant cell death (~30%), and changes in calcium cytosolic levels were observed. As in many other studies (Eggett et al., 2000; Rembach et al., 2004; Benkler et al., 2013; Maier et al., 2013; Liu et al., 2015) authors suggested that, because of their motor neuron origin, the NSC-34 cell line could be used to investigate excitotoxicity mechanisms. However, as reported by Durham et al. (1993), in our study we found limitations in the use of the NSC-34 cell line for neurotoxicity testing. Durham et al. (1993) have evaluated the value of these cells by following exposure of cultures to chemicals known to be neurotoxic for MN. Authors showed that NSC-34 responded to agents that affect voltage-gated ion channels, cytoskeleton organization and axonal transport. However, no electrophysiological evidence was shown, and exposure to glutamate (1 mM) had no effect on cell morphology or potential production. They concluded that the NSC-34 cell line was not a good model to investigate agents that affect synaptic transmission (Durham et al., 1993). Hemendinger et al. (2012) have demonstrated that riluzole in the neurorescue paradigm was unable to reduce cell death induced by neurotoxins that increased intracellular calcium levels independently of ER stress in NSC-34_D_ cells, but they did not provide information on the molecular basis. This finding may potentially be explained by the fact that NSC-34 is not a suitable *in vitro* model to investigate excitotoxicity. All these results together suggest that NSC-34 cells may not be appropriate as an *in vitro* model to study glutamatergic toxicity in motor neuron degeneration. Motor neuronal properties evaluated on differentiated NSC-34 cells and literature findings were summarized in the Appendix (Table A4, see Supplementary Material).

Importantly, the phenotypes of NSC-34 may vary, according to the repeated culture passage or to the origin of the cell line [provided by Dr Neil Cashman (Durham et al., 1993; Maier et al., 2013; Liu et al., 2015) or purchased (Hemendinger et al., 2012)]. So, results may not be reproducible over time, from one laboratory to another, and even within the same laboratory. We cannot exclude the possibility that this contradictory findings may be explained by the differences between basal features of NSC-34 cells, rarely detailed in the studies (Eggett et al., 2000; Rembach et al., 2004; Benkler et al., 2013). There may be various differentiation processes with various media and chemicals known to induce various NSC-34-expressed motor neuron properties, and the culture conditions were not systematically explored to seek optimal toxicity to glutamate. However, in agreement with the literature, in our study we did not find any condition which promoted expression of all glutamatergic properties. The aim of this work was not to focus on optimization of differentiation processes which should involve several parameters (medium composition, differentiation time, time of changing medium, cell density, cell passage, *etc*.) but rather to evaluate the versatility of the NSC-34 cell line to model motor neuron susceptibility to glutamate-induced excitotoxicity. Reliable validation is a crucial step before using a cell line as an *in vitro* model in glutamate-induced excitotoxicity studies. Such validation requires the evaluation of the biochemical and electrophysiological processes of MN that are targets of glutamate such as glutamate receptors and Ca^2+^ influx with a “positive control”.

We could not exclude the possibility that the maturity was insufficient to induce calcium influx in NSC-34_D_ cells under the present experimental conditions. Even if organotypic spinal cord cultures remain the models that closely reflect *in situ* reality, we suggest that the use of primary motor neuron culture is more suitable than NCS-34 cell line to explore the pathogenesis of glutamate-mediated excitotoxicity at the cellular level, in ALS and other motor neuron diseases. However, different authors have shown that NSC-34 cells remain a suitable model of vulnerability to neurotoxins (Maier et al., 2013) and to investigate the effects of cerebrolysin (Keilhoff et al., 2014). NSC-34 cells line is a viable *in vitro* cell model for screening therapeutic candidates against nerve agents (Kanjilal et al., 2014), investigating the expression of transcription factors (Prell et al., 2012) and could thus be a relevant model for some specific experiments.

## Author Contributions

BMH, RF, FP and MG conducted all experiments, analyzed the data and BMH wrote the manuscript. HB, SM, PV, CR and CRA designed the project, analyzed the data and wrote the manuscript. All authors listed, have made substantial, direct and intellectual contribution to the work, and approved it for publication.

## Funding

This work was supported by the “Institut National de la Santé et de la Recherche” INSERM, the University François-Rabelais de Tours, and ARSLA (“Association pour la Recherche sur la Sclérose Latérale Amyotrophique et autres maladies du motoneurone”). BMH was supported by “La Région Centre” with a PhD graduate grant.

## Conflict of Interest Statement

The authors declare that the research was conducted in the absence of any commercial or financial relationships that could be construed as a potential conflict of interest.

## Abbreviations

α-MEM: Alpha-modified Eagle’s medium
ALS: amyotrophic lateral sclerosis
AMPA: α-amino-3-hydroxy-5-methyl-4-isoxazole propionic acid
Ca^2+^: Calcium ion
DAPI: 4,6-diamino-2-phenylindole
DMEM: Dulbecco’s modified Eagle’s medium
FCS: fetal calf serum
KA: kainite
MEM-NEAA: modified Eagle’s medium non-essential amino acids
NSC: Neuroblastoma × Spinal Cord
NSC-34_D_: differentiated NSC-34
MN: motor neurons
NTFs: neurotrophic factors
PBS: phosphate buffered saline
P/S: penicillin/streptomycin
RA: *all-trans* retinoic acid.
