# Supplementary material for: NSC-34 Motor Neuron-Like Cells Are Unsuitable as Experimental Model for Glutamate-Mediated Excitotoxicity
Source: Front Cell Neurosci. 2016 May 9;10:118. doi: 10.3389/fncel.2016.00118 (PMC4860417; doi:10.3389/fncel.2016.00118)
Supplement: Supplementary file 1 [file DataSheet_1.pdf]

Appendix

NSC-34 motor neuron-like cells are unsuitable as experimental model for glutamate-mediated excitotoxicity

Blandine Madji Hounoum[1], Patrick Vourc'h[1,2], Romain Felix[3], Philippe Corcia[1,4], Franck Patin[1,2], Maxime Guéguinou[3], Marie Potier-Cartereau[3], Christophe Vandier[3], Cédric Raoul[5], Christian R Andres[1,2], Sylvie Mavel[1], Hélène Blasco[1,2*]

Table A1. Primer sequences used for RT-PCR p 3

Table A2. Composition of Physiological Saline Solution p 4

Figure A1. RT-PCR on total RNA isolated from mouse brain used as positive control for optimal and specific amplification of glutamate receptor subunits G1uA1-4 GluN1, GluN2A-D and GluN3A p 5

Figure A2. SDS-PAGE western blot analysis of lysates from mice brain (positive control), differentiated NSC-34 (NSC-34_D) and non-differentiated NSC-34 carried out using antibodies against GluN2A glutamate receptor subunit p 6

Table A3. Relative quantification of glutamate receptor subunit gene expression in NSC-$34_{D.}$ – (no expression), + to +++ (weak to high expression) p 7

Figure A3: (A) Morphological characterization of primary motor neuron culture. Immunostaining of motor neuron cells for βIII-tubulin indicated in green, p75NTR in red, nuclei stained with DAPI in blue, and merged channel (scale bar: 50 μM). (B) Glutamate dependent calcium entry into motor neurons at 5, 12 and 13 DIV (Days In Vitro). Representative fluorescence measurement of Ca2+ entry after acute application of glutamate (100 μM) in motor neurons. Glutamate was added at the time indicated by the arrow.

Measurements of intracellular calcium were performed independently at 5, 12 and 13 DIV. All results for each time point (5, 12 and 13 DIV) were an average of three independent experiments (coverslips) with at least five cells per coverslip analyzed. P8

Table A4: Motor neuronal properties evaluated on NSC-34 cells compared to motor neurons in our study and the literature findings. MN, motor neurons; NSC-34_D, differentiated NSC-34 cells, + to ++++ (weak to high) p9

Table A1: Primer sequences used for conventional RT-PCR and RT-qPCR

Gene	Forward primer (5'-3')	Reverse primer (5'-3')	Amplified fragment (bp)
GluA1	ACCACTACATCCTCGCCAAC	TCACTTGTCCTCCACTGCTG	145
GluA2	ATTTCGGGTAGGGATGGTTC	AAAACTGGGAGCAGAAAGCA	116
GluA3	CACCAACCAGAACACCACTG	GCATACACCCCTCTGGAGAA	123
GluA4	CATTTCACCCAGATCCCTGT	GCCAGGTCTTCTGCACTTTC	150
GluN1	CGGCTCTTGGAAGATACAGC	GTGGGAGTGAAGTGGTCGTT	156
GluN2A	TTGTGGTGATCGTGCTGAAT	CTCCAAGGTGACAATGCTGA	155
GluN2B	TCCGAAGCTGGTGATAATCC	TCCTCCAAGGTAACGATGCT	162
GluN2C	AACCACACCTTCAGCAGCG	GACTTCTTGCCCTTGGTGAG	464
GluN2D	CGATGGCGTCTGGAATGG	AGATGAAAACTGTGACGGCG	265
GluN3A	CCGCGGGATGCCCTACTGTTC	CCAGTTGTTCATGGTCAGGAT	417
ChAT	CCAACCAAGCCAAGCAATCT	AAGGATAGGGGAGCAGCAACAA	114
P75	CAACCAGACCGTGTGTGAAC	CCAGTCTCCTCGTCCTGGTA	183
Actin	TTGCTGACAGGATGCAGAAG	TGATCCACATCTGCTGGAAG	147

Table A2: Composition of Physiological Saline Solution

**Composition**	**Solution Ca^{2+} 2 mM**
**NaCl**	140 mM
**KCl**	4 mM
**$MgCl_2$**	1 mM
**$CaCl_2$**	2 mM
**Hepes**	10 mM
**Glucose**	11.1 mM
**pH (adjusted with NaOHaq)**	7.4
	total volume adjusted with H_2O

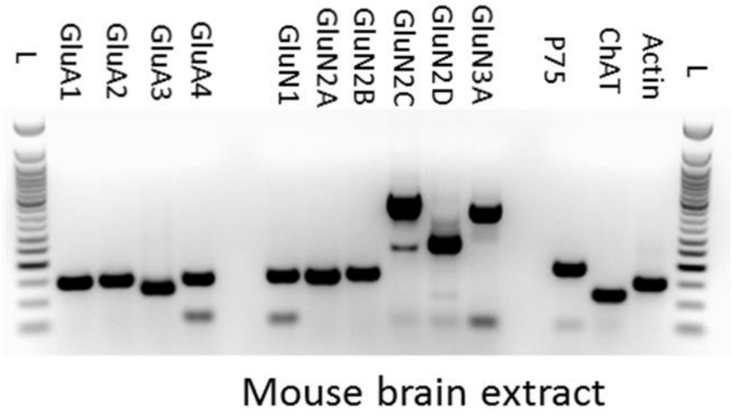

Fig. A1. RT-PCR on total RNA isolated from mouse brain used as positive control for optimal and specific amplification of glutamate receptor subunits GluA1, GluA2, GluA3, GluA4, GluN1, GluN2A, GluN2B, GluN2C, GluN2D and GluN3A.

Figure A2: SDS-PAGE western blot analysis of lysates from mice brain (positive control), differentiated NSC-34 ($NSC\text{-}34_D$) and non-differentiated NSC-34 carried out using antibodies against GluN2A glutamate receptor subunit.

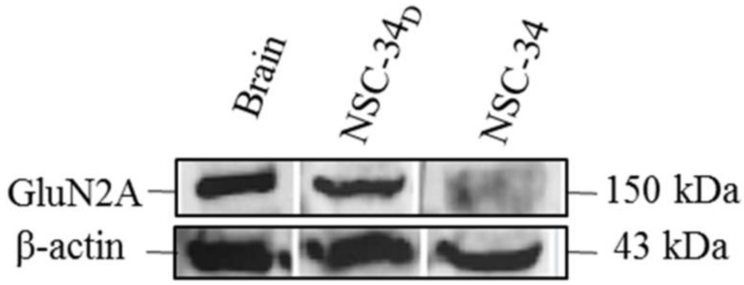

Table A3: Relative quantification of glutamate receptor subunit gene expression in NSC-34$_{D}$.

– (no expression), + to +++ (weak to high expression)

				NSC-34$_{D}$					
				Ham's F12		α-MEM		DMEM	
	Mouse brain	MN	NSC-34	+RA	-RA	+RA	-RA	+RA	-RA
GluA1	+++	+++	+	+	+	+	+	+	-
GluA2	+++	+++	-	+	+	+	-	+	-
GluA3	+++	+++	-	-	+	+	-	-	-
GluA4	+++	+++	-	+	+	+	+++	++	+++
GluN1	+++	+++	+	++	+++	+++	+++	++	+++
GluN2A	+++	+++	+	-	+++	+	++	-	++
GluN2B	+++	+++	-	-	-	-	+	-	-
GluN2C	+++	+++	-	-	-	-	-	-	-
GluN2D	+++	+++	+	++	+++	+++	++	++	+++
GluN3A	+++	+++	-	-	-	-	-	-	-
ChAT	+++	++	++	++	+++	+++	+++	+++	+++
P75	+++	+++	++	++	+++	++	+++	++	+++
Actin	+++	+++	++	++	+++	+++	+++	+++	+++

Figure A3: (A) Morphological characterization of primary motor neuron culture. Immunostaining of motor neuron cells for βIII-tubulin indicated in green, p75NTR in red, nuclei stained with DAPI in blue, and merged channel (scale bar: 50 μM). (B) Glutamate dependent calcium entry into motor neurons at 5, 12 and 13 DIV (Days In Vitro). Representative fluorescence measurement of Ca2+ entry after acute application of glutamate (100 μM) in motor neurons. Glutamate was added at the time indicated by the arrow. Measurements of intracellular calcium were performed independently at 5, 12 and 13 DIV. All results for each time point (5, 12 and 13 DIV) were an average of three independent experiments (coverslips) with at least five cells per coverslip analyzed.

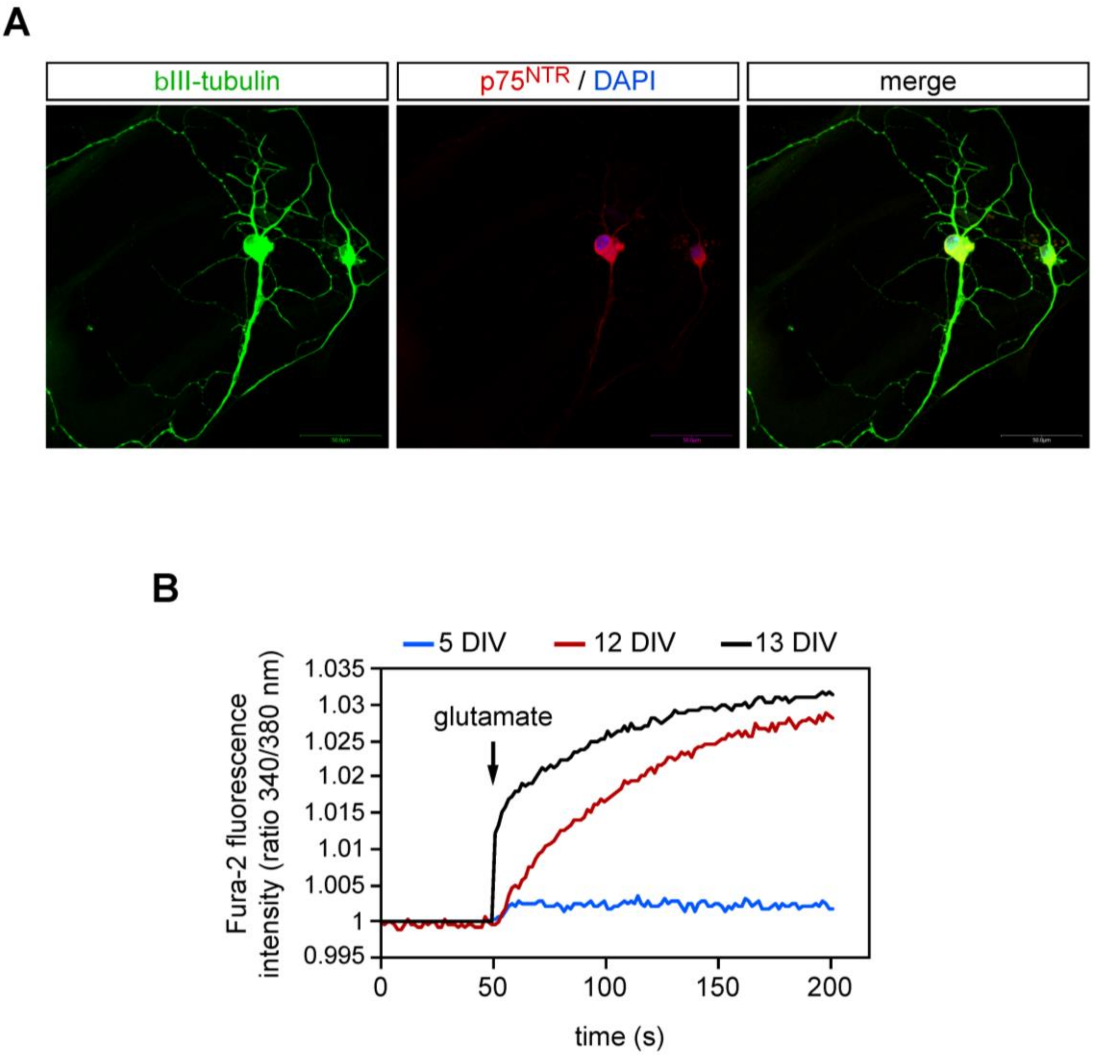

Table A4: Motor neuronal properties evaluated on NSC-34 cells compared to motor neurons in our study and the literature findings about NSC-34 cells. MN, motor neurons; NSC-34_D, differentiated NSC-34 cells, + to ++++ (weak to high).

MN properties		MN	NSC-34_D	NSC-34	Literature
Growth factor receptors	P75NTR	++	+++	++	Turner et al. (2004), Matusica et al. (2008)
Cholinergic markers	ChAT	+	+	+	Rembach et al. (2004), Maier et al. (2013)
Glutamate receptor subunits	GluA1	+++	+	+	Eggett et al. (2000), Rembach et al (2004)
	GluA2	+++	+	-	Eggett et al. (2000), Rembach et al (2004); Liu et al (2015)
	GluA3	+++	+	-	Eggett et al. (2000), Rembach et al (2004)
	GluA4	+++	+	-	Eggett et al. (2000), Rembach et al (2004)
	GluN1	+++	++++	+	Eggett et al. (2000)
	GluN2A	+++	++	+	Eggett et al. (2000)
	GluN2B	+++	-	-	Eggett et al. (2000)
	GluN2D	+++	++++	+	Eggett et al. (2000)
Ca^{2+} influx		++++	+	-	Eggett et al. (2000), Liu et al (2015)
Glutamate sensitivity		++++	+	-	Maier et al. (2013), Durham et al. 1992, Liu et al (2015)
